# Thermal Analysis and Prediction Methods for Temperature Distribution of Slab Track Using Meteorological Data

**DOI:** 10.3390/s22176345

**Published:** 2022-08-23

**Authors:** Qiangqiang Zhang, Gonglian Dai, Yu Tang

**Affiliations:** 1 School of Civil Engineering, Central South University, Changsha 410083, China; 2 School of Resources and Safety Engineering, Central South University, Changsha 410083, China

**Keywords:** scale model test, slab track, thermal analysis, theoretical study, meteorological data

## Abstract

The structural temperature distribution, especially temperature difference caused by solar radiation, has a great impact on the deformation and curvature of the concrete slab tracks of high-speed railways. Previous studies mainly focused on the temperature prediction of slab tracks, while how the temperature distribution is affected by environmental conditions has been rarely investigated. Based on the integral transformation method, this work presents an analytical method to determine and decompose the temperature distribution of the concrete slab track. A field temperature test of a half-scaled specimen of concrete slab track was conducted to validate the developed methodology. In the proposed method, we decompose the temperature distribution of the slab track into an initial temperature component and a boundary temperature component. Then, the boundary temperature components caused by solar radiation and atmospheric temperature are investigated, respectively. The results show that the solar radiation plays a significant role in the nonlinear temperature distribution, while the atmospheric temperature has little effect. By contrast, the temperature change in the slab surface resulting from the atmospheric temperature accounts on average for only 5% in the hot weather condition. The proposed method establishes a relation between the structural temperature and meteorological parameters (i.e., the solar radiation and atmospheric temperature). Consequently, the temperature distribution of the concrete slab track is predicted via the meteorological parameters.

## 1. Introduction

**Figure 1:**
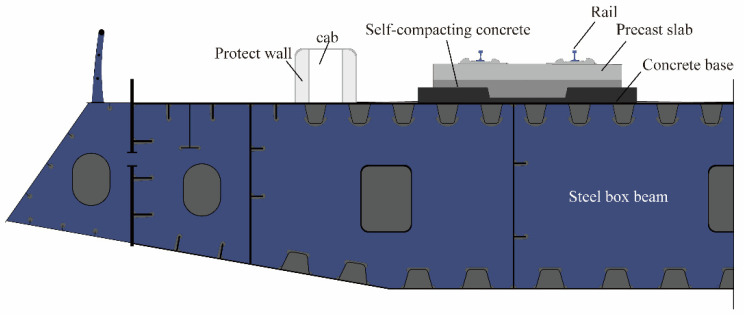
A concrete slab track on a steel box girder section.

Slab tracks have been widely used in high-speed railways because of their advantages of high stability and smoothness. By 2020, nearly 29,000 km of slab tracks had been built in China, which accounts for more than 80% of all slab tracks in the world [1]. At present, there are three types of China Railway Track System (CRTS) slab tracks, namely, CRTS-I, CRTS-Ⅱ, and CRTS-III. In particular, the CRTS-Ⅲ slab track, which is technically improved from the German Bögl slab track, has been used in new railway lines. Among these, a CRTS-Ⅲ slab track laid over a steel bridge was first built, as shown in Figure 1, which consists of a precast slab, self-compacting concrete, and a concrete base, and can be defined as a multilayered structure. Different from slab tracks on concrete bridges, the heat flow through the slab track to the steel bridge is easier and faster due to the limited height of the concrete members of the bridge-track system. Consequently, this may lead to a larger temperature difference. The novel construction of a slab track on a steel bridge requires more attention to be paid to the temperature evolution of concrete slab tracks under the environmental conditions.

Slab tracks are affected by environmental conditions, such as solar radiation, air temperature, and wind, and can hypothetically produce two periodic thermal actions. First, the seasonal changing temperature may cause the overall concrete slab to rise or drop, and its macro-performance consists of the expansion and contraction deformation. Second, the daily solar radiation and air temperature variations may lead to a temperature difference that causes bending deformation in the slab track [2,3,4]. Considering some temperature-induced damage problems in other slab tracks, the CRTS-III slab track and France’s New Ballastless Track have considered the temperature effect on slab tracks [5]. In addition, some physical methods to reduce the slab temperature have also been tested [6,7,8]. These involve coating the slab surface with composite materials to reduce the radiation absorption or increase the reflected radiation.

Temperature is one of the most critical parameters related to the behavior and response of slab tracks. Temperature tests of slab tracks in the laboratory under specific conditions have attracted more attention. Yang et al. [2] analyzed the temperature distribution of the slab track based on a full-scale temperature test and found that a long-term daily mean air temperature and stronger solar radiation caused the whole slab temperature to rise under continuous hot weather conditions. Zhang et al. [9] developed a 1:4 scaled laboratory test for CRTS-II slab track, which was used to quantify the stresses in the various components of the track system resulting from sudden temperature variations. Zhong et al. [4] investigated the impact of the daily air temperature on the interface stress of a full-scale specimen of CRTS-II slab track in the construction stage. Zhou et al. [10] compared the effect of the constraint conditions on the temperature distribution of a slab track in two 1:4 scaled specimens and found that the fixed constraint condition decelerated the temperature transfer of the track slab to the cement asphalt mortar. Zhou et al. [11,12] carried out a temperature test of a 1:4 scaled specimen for a CRTS-II slab track on three simply-supported box girders with a heat device and then analyzed the distribution of the three-dimensional thermal fields in the slab. They also found that the strains in the track structure increased nonlinearly with the environmental temperature increase [13]. Although there were some satisfactory conclusions to be drawn from the temperature test of slab tracks, there is currently a lack of research on the different effects of solar radiation and air temperature on the temperature distribution of slab tracks on a steel bridge.

Slab tracks built in a natural environment undergo a complicated heat transfer, and it is theoretically necessary to investigate the temperature evolution of slab tracks subjected to environmental thermal loads. Ou et al. [14] investigated the temperature distribution of CRTS-II slab track during four seasons based on a simplified solution of heat conduction. It was found that air temperature was one of main factors affecting the temperature distribution inside the track structure, and the temperature gradient was biggest in the summer. Liu et al. [15] offered a simplified solution of the thermal field of concrete slab track to reveal the relation between temperature distributions and environmental conditions. The results showed that the combined action of the mean air temperature and solar radiation impacted the overall concrete slab temperature, and the air temperature amplitude greatly influenced the temperature gradient. Zeng et al. [16] provided an analytical solution to a semi-infinite thermal field model of concrete slab track, which effectively predicted the temperature gradient of the track structure in different cities in China. Riding et al. [17] calculated the thermal field of concrete structures using three recommended methods in specifications and proved that the error based on heat conduction equations was the smallest. In summary, previous researchers have sought a simplified method for solving the heat problem of slab tracks under external environmental conditions, which is convenient for demonstrating the temperature evolution of slab tracks in one day. However, they ignore the coupling actions of solar radiation and air temperature on the temperature distribution of slab tracks. Therefore, it is necessary to find an analytical method to distinguish the different effects of solar radiation from air temperature on the temperature evolution of slab tracks.

As the slab track system is similar with the multilayered concrete pavement, the analytical method used for concrete pavement temperature can be consulted in [18,19,20,21,22,23]. The Green’s function method was used for the analytical solution of thermal field in the multilayered pavement [18]. A one-dimensional temperature model of the pavement under site conditions was solved by the Laplace transform method [19]. The heat problem with the measured surface temperature was solved without considering the environmental conditions [24]. Compared to the semi-infinite pavement system, the slab track is a three-dimensional thermal field due to the finite size of the structure. The different types and numbers of boundary conditions make the governing equations hard to be solved by analytical methods.

Focusing on the different effects of solar radiation and air temperature on concrete slab tracks, this paper proposes an analytical prediction method for the temperature distribution of concrete slab tracks via meteorological parameters. The structure of this paper is as follows. First, an experimental program of a half-scaled specimen of a concrete slab track was described for an outdoor temperature test. Then, a one-dimensional temperature model of the concrete slab was developed while considering the meteorological parameters and solved based on the integral transformation method. The calculation accuracy was approved using other methods. Combined with the solution, the temperature distribution in a generic concrete section is decomposed theoretically and the different effects of solar radiation and air temperature on the time–space temperature distribution in slab tracks is discussed.

## 2. Experimental Setup

### 2.1. Specimen Design

**Figure 2:**
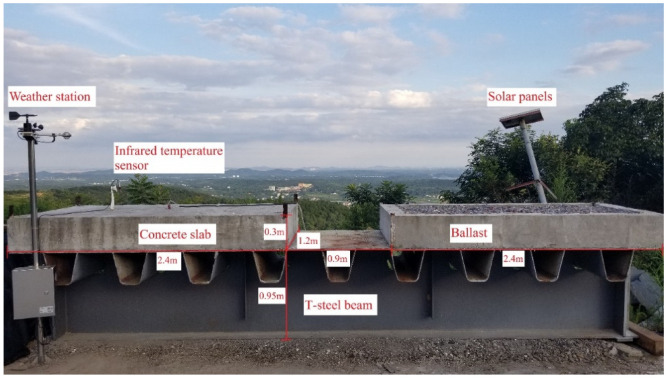
Temperature test of track structures on a T-steel beam segment.

**Figure 3:**
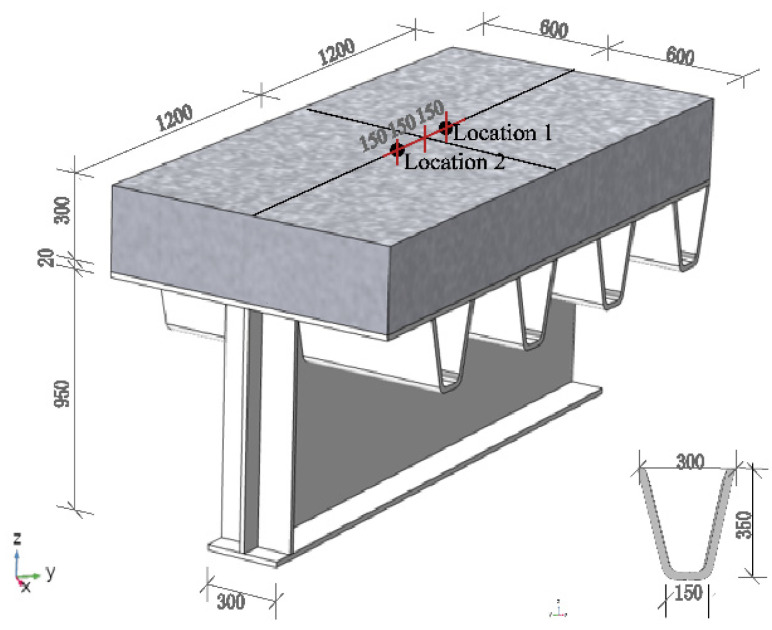
Geometric dimensions and measuring positions of the testing specimen (unit: mm).

The test site is located on the top of a hillside in Changsha, China (112° E, 27° N), with an altitude of 150 m. The region has a subtropical climate with an annual average temperature of 28 °C. Before the construction, a suitable location was chosen in an open area without shade to meet the requirement of natural solar radiation thermal loads. The temperature test of the track structure is shown in Figure 2. The experimental beam segment is 1.2 m in width, 5.7 m in length, and 0.95 m in height. The thickness of the flange and web of the steel beam are 20 mm and 15 mm, respectively. The concrete slab was cast to the left side of the steel beam segment, while the other side was manufactured as a ballast track structure. Since the objective of this paper was to investigate the thermal field of a concrete slab track structure, only the monitoring results of the left side of the specimen were used, as shown in Figure 3.

In general, the temperature change in the concrete members along the longitudinal section is small [15,16,25], so concrete slab tracks can be reduced to a half-scaled concrete slab specimen, with a specific size of 2.4 m × 1.2 m × 0.3 m. The detailed dimension of the temperature test specimen is shown in Figure 3. The concrete slab is made of ordinary cement, water, crushed sand, and gravel with a mixing ratio of 210, 355, 703, and 887 Kg·m^−3^, respectively. The beam segment is made of Q235b steel with a grayish color coating. In addition, to prevent the slippage of the concrete slab resulting from the thermal action, vertical reinforcements with a diameter of 20 mm were welded on the top plate of the steel beam at a spacing of 1.2 m.

### 2.2. Arrangement of Temperature Sensors

**Figure 4:**
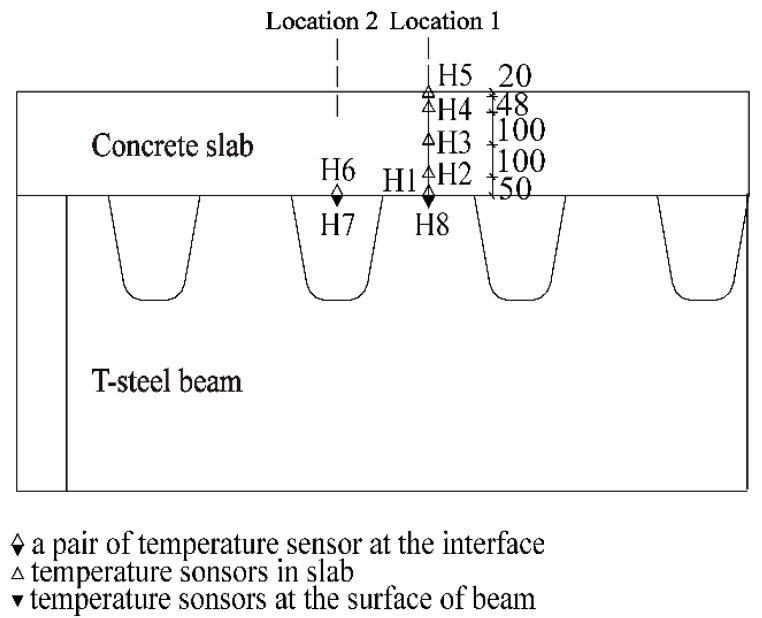
Distributions of temperature sensors (unit: mm).

In this study, the test is to measure the vertical temperature distribution in the concrete slab. To prevent the influence of the transverse heat flow on the measuring point, the center location of the concrete slab was selected as the test section, namely, Location 1 (Figure 3). Five temperature sensors (H1–H5) were embedded in the concrete slab along the depth at Location 1. The distribution of all temperature sensors is clearly shown in Figure 4.

The temperature of the contact interface was measured to obtain the bottom boundary condition. Two pairs of measuring points were arranged at different locations of the contact interface. The one pair of measuring points with the numbers H1 and H8 was in Location 1 and another point with the numbers H6 and H7 was in Location 2. Points H7 and H8 were pasted on the bottom surface of the top plate of the steel beam along Locations 1 and 2. 

**Figure 5:**
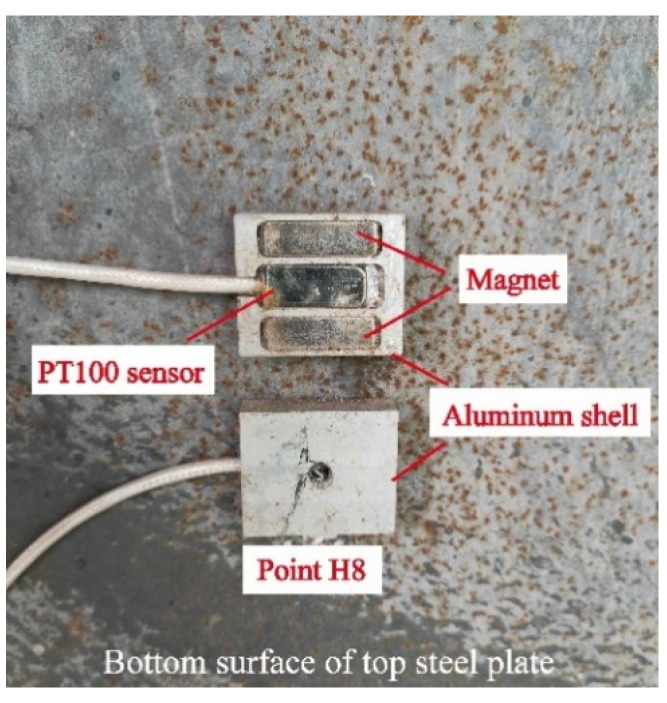
The exterior sensor with an aluminum square shell.

The PT 100 platinum thermal sensor of 30 mm in length, 8 mm in width, and 4 mm in thickness was used for the measurement. The working range of the sensor is −50~200 °C, and the testing precision is ±0.2 °C. To prevent the influence of reflected radiation on the exterior sensor (H7 and H8), an aluminum square shell was constructed to cover the sensor, as shown in Figure 5.

### 2.3. Meteorological Parameters

**Figure 6:**
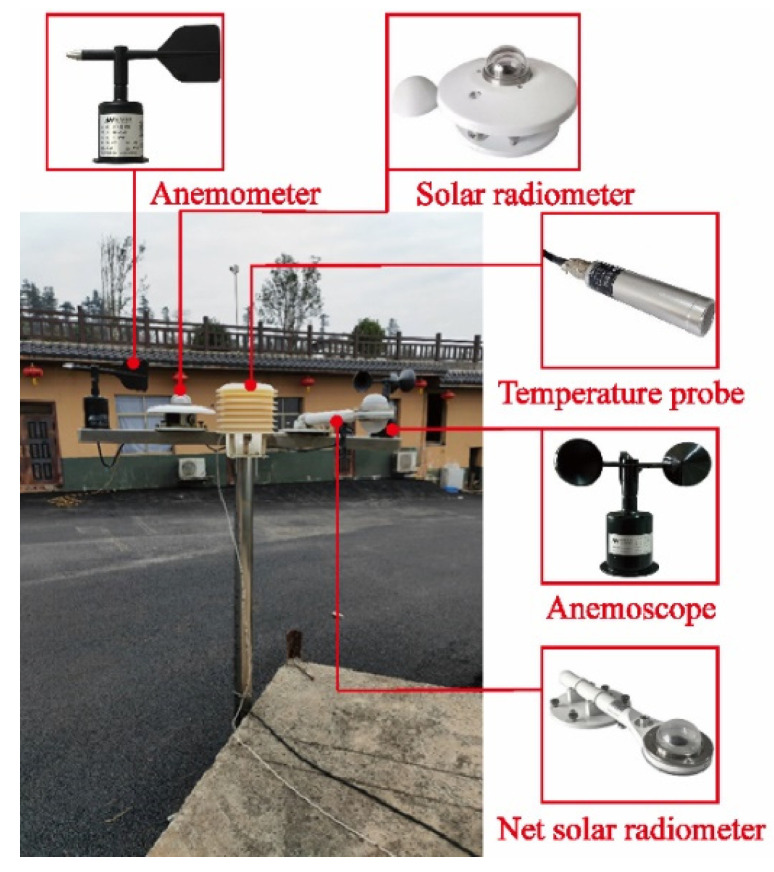
Environment monitoring system.

The monitoring meteorological parameter is essential for the prediction and analysis methods [26]. For accurate boundary conditions of the thermal field, a weather station was built at the experimental site, as shown in Figure 6. The weather station monitors five meteorological parameters. The solar radiometer is used to observe the total solar radiation intensity on the horizontal plane. The temperature probe in the instrument shelter records the air temperature. The anemometer and anemoscope are applied to measure wind direction and wind speed. The net solar radiometer is used to observe the instantaneous heat budget. 

A solar-powered system was adopted to provide energy for the structural temperature and meteorological monitoring system. The data were collected every 30 min by an automatic acquisition instrument since 1 October 2019. 

## 3. Analytical Prediction Method

### 3.1. Analytical Solution of a One-Dimensional Temperature Distribution 

**Figure 7:**
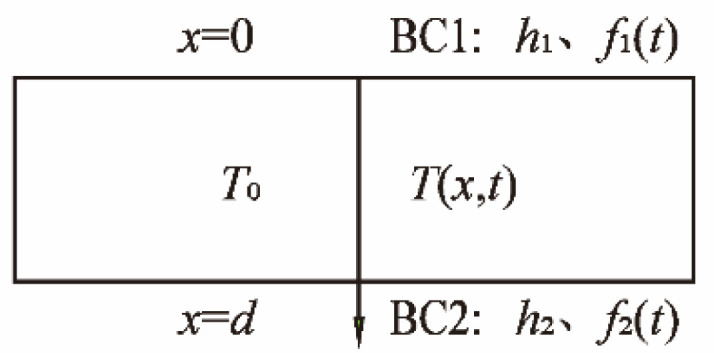
One-dimensional model of the temperature distribution.

In practical engineering, the vertical temperature distribution of a slab track is the main consideration, especially the most unfavorable temperature distribution under extreme environmental conditions. The calculated result of the multi-dimensional temperature model was close to that of the one-dimensional model [25]. For mathematical simplicity, we only consider a one-dimensional heat conduction problem with double convection boundaries in the finite region, representing change through depth, as shown in Figure 7. 

In Figure 7, The surfaces BC1 and BC2 of the slab are heated by the convection of two ambient fluids: *f*_1_(*t*) and *f*_2_(*t*). It is necessary to develop a unique function for the boundary condition variables *f*_1_(*t*) and *f*_2_(*t*) when applying analytical methods. A third-order polynomial function had been used to achieve approximation [19]. For a special case of the boundary conditions of a period convection in this problem, the mathematical model of the ambient fluid temperature is assumed to be a cosine function:

(1) $$f_{i}(t)=T_{i,a}-T_{i,b}\cos(w_{i}(t-\delta_{i})),t\in (t_{1}∼t_{2})$$

where *i* is the number of boundary surfaces, *T_i_*_,a_ and *T_i_*_,a_ are the average temperature and amplitude of the fluid medium, respectively, *w_i_* and *δ_i_* are the frequency and phase of the function, respectively, and *t*_1_ and *t*_2_ are the sunrise and sunset time, respectively.

As there is no internal heat source in the temperature model, the heat conduction equation is expressed as Equation (2). The boundary conditions of surfaces BC1 and BC2 are considered by Equations (3) and (4), and Equation (5) gives the initial condition of this problem:

(2) $$\alpha \frac{\partial^{2}T\left(x,t\right)}{\partial x^{2}}=\frac{\partial T\left(x,t\right)}{\partial t}\;\mathrm{in}\;0<x<d,\;t_{1}<0<t_{2}$$

(3) $$\mathrm{BC}1:\;-k\frac{\partial T\left(x,t\right)}{\partial x}+h_{1}T\left(x,t\right)=h_{1}f_{1}(t)\;,\;x=0$$

(4) $$\mathrm{BC}2:\;k\frac{\partial T\left(x,t\right)}{\partial x}+h_{2}T\left(x,t\right)=h_{2}f_{2}(t)\;,\;x=d$$

(5) $$\mathrm{IC}:\;T(x,t_{1})=T_{0}$$

where α is the thermal diffusivity (m^2^·s^−1^), *k* is the thermal conductivity (W m^−1^·K^−1^), *h*_1_ and *h*_2_ are heat transfer coefficients at different surfaces (W∙m^−2^·K^−1^), and *T*(*x*, *t*) is the temperature (°C) at an arbitrary point *x* at any time *t*.

To make the initial temperature *T*(*x*, *t*_1_) be equal to zero, the following temperature variable is introduced:

(6) $$\theta \left(x,t\right)=T\left(x,t\right)-T_{0}$$

Then, Equations (3)–(5) are transformed as:

(7) $$\mathrm{BC}1:\;-k\frac{\partial \theta \left(x,t\right)}{\partial x}+h_{1}\theta \left(x,t\right)=h_{1}\left(f_{1}(t)-T\left(x,t_{1}\right)\right)\;$$

(8) $$\mathrm{BC}2:\;k\frac{\partial \theta \left(x,t\right)}{\partial x}+h_{2}\theta \left(x,t\right)=h_{2}\left(f_{2}(t)-T\left(x,t_{1}\right)\right)$$

(9) $$\mathrm{IC}:\;\theta \left(x,t_{1}\right)=0$$

To solve the aforementioned heat conduction problem, the integral transform pair for the function *θ*(*x*, *t*) with respect to the *x* variable is constructed based on the integral transformation method [27]:

(10) $$\theta (x,t)=\sum_{n=1}^{\infty }\frac{X(\beta_{n},x^{\prime })}{N(\beta_{n})}\bar{\theta }(\beta_{n},t)$$

(11) $$\bar{\theta }\left(\beta_{n},t\right)=\int_{0}^{d}X\left(\beta_{n},x^{\prime }\right)\theta \left(x^{\prime },t\right)dx^{\prime }$$

where *N*(*β_n_*) is the norm and *X*(*β_n_, t*) is the eigenfunction. There are infinite norms and eigenfunctions for the eigenvalues *β_n_*. By the application of the transformation (Equations (10) and (11)), the general solution of Equation (2) is in the following form [27]:

(12) $$\theta (x,t)=\sum_{n=1}^{\infty }\frac{X(\beta_{n},x^{\prime })}{N(\beta_{n})}e^{-\alpha \beta_{n}{}^{2}t}\int_{0}^{t}e^{\alpha \beta_{n}{}^{2}t^{\prime }}A(\beta_{n},t^{\prime })dt^{\prime }$$

where

(13) $$N(\beta_{n})=\int_{0}^{d}{\left[X(\beta_{n},x^{\prime })\right]}^{2}dx^{\prime }$$

(14) $$A\left(\beta_{n},t^{\prime }\right)=\frac{\alpha }{k}\left[X\left(\beta_{n},0\right)h_{1}f_{1}\left(t^{\prime }\right)+X\left(\beta_{n},d\right)h_{2}f_{2}\left(t^{\prime }\right)\right]$$

For the boundary value problem with a double-convective boundary condition, the eigenfunction and transcendental function are expressed as follows [27]:

(15) $$X\left(\beta_{n},x\right)=\beta_{n}\cos\left(\beta_{n},x\right)+H_{1}\sin\left(\beta_{n},x\right)$$

(16) $$\tan\left(\beta_{n}d\right)=\frac{\left(H_{1}+H_{2}\right)\beta_{n}}{\beta_{n}{}^{2}-H_{1}H_{2}}$$

According to temperature variable *θ*(*x*, *t*), the function of ambient fluid temperature (Equation (1)) becomes:

(17) $$f_{i}(t)=\Delta T_{f,i}+T_{f,i}(t)i=1,2$$

where $\Delta T_{f,i}=T_{i,a}-T_{0}$ and $T_{f,i}(t)=-T_{i,b}\cos(w_{i}(t-\delta_{i}))$.

The expression (Equation (17)) is introduced into the general solution (Equation (12)). Then, the definite integral can be evaluated, and the solution is expressed as:

(18) $$\theta \left(x,t\right)=\sum_{i=1}^{2}\sum_{n=1}^{\infty }\frac{X\left(\beta_{n},x\right)}{N\left(\beta_{n}\right)}\frac{h_{i}X\left(\beta_{n},x_{i}\right)}{k\beta_{n}{}^{2}}\cos\left(w_{i}\varphi_{i,n}\right)\left\{T_{f,i}\left(t-\varphi_{i,n}\right)+\frac{\Delta T_{f,i}}{\cos\left(w_{i}\varphi_{i,n}\right)}-\left[T_{f,i}\left(t_{1}-\varphi_{i,n}\right)\right.\right.+\left.\left.\frac{\Delta T_{f,i}}{\cos\left(w_{i}\varphi_{i,n}\right)}\right]e^{-\alpha \beta_{n}{}^{2}\left(t-t_{1}\right)}\right\}$$

By substituting Equation (6) into Equation (18), the analytical solution of the one-dimensional original heat conduction problem is obtained, as shown in Equation (19):

(19) $$T\left(x,t\right)=T_{0}+\sum_{i=1}^{2}\sum_{n=1}^{\infty }C_{i,n}X\left(\beta_{n,}x\right)[f_{i,n}\left(t\right)-e^{-\alpha \beta_{n}{}^{2}\left(t-t_{1}\right)}f_{i,n}\left(t_{1}\right)]$$

where it is defined that:

(20) $$f_{i,n}\left(t\right)=\frac{\Delta T_{f,i}}{\cos\left(w_{i}\varphi_{i,n}\right)}+T_{f,i}\left(t-\varphi_{i,n}\right)$$

(21) $$C_{i,n}=\frac{H_{i}\cos\left(w_{i}\varphi_{i,n}\right)X\left(\beta_{n},x_{i}\right)}{N\left(\beta_{n}\right)\beta_{n}{}^{2}}$$

(22) $$\cos\left(w_{i}\varphi_{i,n}\right)=\frac{\alpha \beta_{n}{}^{2}}{\sqrt{{\left(\alpha \beta_{n}{}^{2}\right)}^{2}+w_{i}{}^{2}}}$$

In Equation (19), the temperature *T*(*x*, *t*) mainly consists of a superposition expression of the boundary temperature term. The analytical solution is a parametric formula, which benefits discussing the effect of the dimensional or environmental parameters. When the initial temperature *T*_0_ is determined, the calculation accuracy of the temperature field is only related to the series expression. The larger the series expansion term *n*, the higher the accuracy.

### 3.2. Temperature Distribution Decomposition

**Figure 8:**
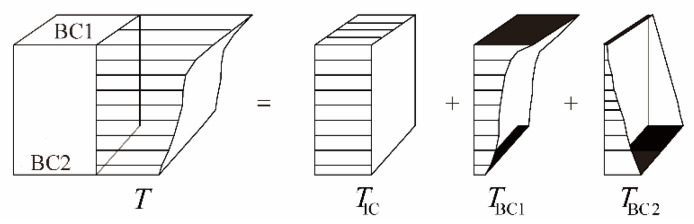
Temperature decomposition of concrete slab.

In the analytical solution, the temperature *T*(*x*, *t*) depends on the initial temperature term and the boundary temperature term. According to the linear superposition principle, the temperature distribution caused by boundary layers at the *i*th boundary can be obtained with decomposing Equation (19). As shown in Figure 8, given an arbitrary thermal field of a slab section with two boundaries, the total thermal field is the sum of these three contributions (see Equation (23)).

(23) $$T=T_{\mathrm{IC}}+T_{\mathrm{BC}}{}_{1}+T_{\mathrm{BC}}{}_{2}$$

The initial temperature component *T*_IC_ is the temperature distribution of the slab at the initial time. The temperature gradient is quite small for a thin slab. The initial temperature distribution is assumed to be linear. Then, the component *T*_IC_ is the area weighted average value of the thermal field:

(24) $$T_{\mathrm{IC}}=T_{0}\left(x,0\right)$$

The boundary temperature components *T*_BC1_ and *T*_BC2_ are generated by boundary surfaces BC1 and BC2, respectively. The component *T*_BC*i*_ (*x*, *t*) consists of the periodic function term and exponential attenuation term, as shown in Equation (25):

(25) $$T_{\mathrm{BC}}{}_{i}\left(x,t\right)=\sum_{n=1}^{\infty }C_{i,n}X\left(\beta_{n,}x\right)\left[f_{i,n}\left(t\right)-e^{-\alpha \beta_{n}{}^{2}\left(t-t_{1}\right)}f_{i,n}\left(t_{1}\right)\right]$$

The temperature decomposition has the advantage of investigating the different actions of the solar radiation and the air temperature on the vertical temperature distribution of slab track. Thereby, the effect of different boundary conditions on the thermal field can be obtained.

### 3.3. The Method of Dealing with Meteorological Parameters

**Figure 9:**
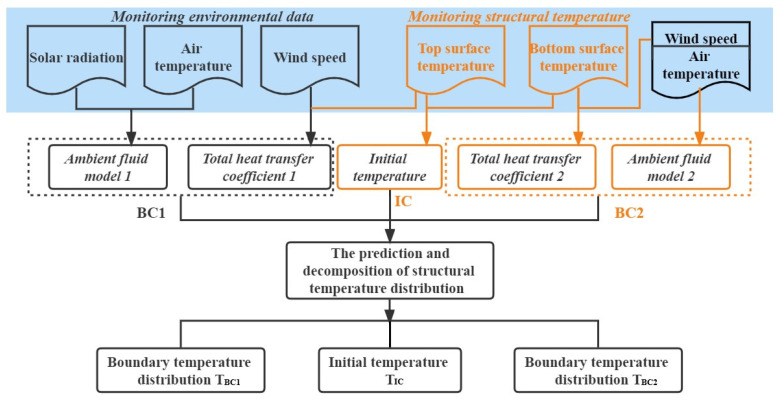
Flowchart of the analytical method using meteorological parameters.

The site environmental conditions are considered and the procedure for dealing with meteorological parameters is illustrated. The main steps of the analytical prediction method for the concrete slab temperature distribution are summarized in Figure 9.

Stage 1: Obtain the site meteorological data for every hour on a sunny and cloudless day, mainly including three meteorological parameters, namely, solar radiation, wind speed, and air temperature. The treatment of the meteorological data is described in Section 3.3.1.

In particular, the air temperature variation is not wide in the continuous sunny weather condition, which is beneficial for the establishment of the ambient fluid model 2 and the initial temperature distribution. Besides, the regular solar radiation can better establish the ambient fluid model 1. The wind speed is mainly used to calculate the heat transfer coefficient, which is not an affecting parameter.

Stage 2: Establish the boundary condition functions *f*_1_(t) and *f*_1_(t) of the concrete slab according to the meteorological parameters in Section 3.3.2. The mathematical model is a cosine function (Equation (1)).

Stage 3: Measure the top and bottom surface temperatures of the slab to develop the initial condition (IC) described in Section 3.3.3. For the thin concrete slab, the initial condition is a constant initial temperature.

Stage 4: Calculate the total heat transfer coefficient by the formula of the radiation heat transfer and convection heat transfer in Section 3.3.4. The coefficient of the radiation heat transfer is calculated approximately using Equation (28).

Stage 5: Apply the analytical solution to obtain the temperature distribution in Section 3.1. 

Stage 6: Use the temperature decomposition method to obtain the temperature components in Section 3.2.

#### 3.3.1. Equivalent Radiation Temperature

**Figure 10:**
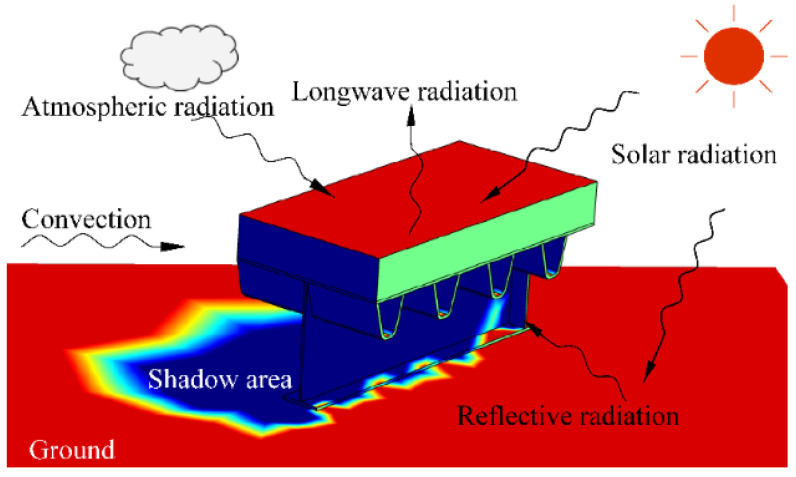
Mechanisms of heat transfer.

In the daytime, the structure absorbs heat from the solar radiation and the atmospheric radiation. Meanwhile, the structure releases heat to the exterior environment through the convection and longwave radiation. The main processes of heat exchange are split in radiation, convection, and conduction, as shown in Figure 10. 

Taking the heat exchange at the top surface (BC1) of the concrete slab as an example, the general equation for net heat transfer is written as:

(26) $$\begin{array}{ll} q & =\gamma q_{s}-h_{c}\left(T_{u}-T_{a}\right)-\varepsilon C_{0}\left(T_{u}{}^{4}-T_{a}{}^{4}\right)\\ & =\gamma q_{s}-\left(h_{c}+h_{r}\right)\left(T_{u}-T_{a}\right) \end{array}$$

where *q*_s_ is the total solar radiation on a plane, which is gained from the measured solar radiation on a horizontal plane or the empirical models of solar radiation, *γ* is the solar absorptivity coefficient of the concrete slab and is taken as 0.5 [14], *ε* is the emissivity of the surface, *C*_0_ is the Stefan–Boltzmann constant (5.67 × 10^−8^ W∙m^−2^∙K^−4^), *T*_u_ and *T*_a_ are the surface and fluid temperatures (K), respectively, and *h*_c_ is the coefficient of the convection heat transfer (W∙m^2^·K^−1^). *h*_c_ is a function of wind speed 𝑣 (m∙s^−1^) and is expressed as [14,15]:

(27) $$h_{c}=4v+5.7$$

The variable *h*_r_ is the coefficient of the radiation heat transfer (W∙m^2^·K^−1^) calculated by the radiation heat transfer Equation (28).

(28) $$h_{r}=\varepsilon C_{0}\left(T_{u}^{2}+T_{a}{}^{2}\right)\left(T_{u}+T_{a}\right)$$

The net heat conduction equation (Equation (26)) is simplified to the boundary condition of the third type by substituting into Equations (27) and (28), which is expressed as:

(29) $$q=-h\left[T_{u}-\left(T_{a}+\frac{\gamma q_{s}}{h}\right)\right]$$

where *h* is the total heat transfer coefficient (W∙m^−2^·K^−1^) and *h = h*_c_ + *h*_r_.

The combined action of the solar radiation and atmospheric temperature on concrete slab surface is considered as the equivalent radiation temperature [19]. The assumed temperature *T*_e_ is obtained from Equation (29), giving:

(30) $$T_{e}=T_{a}+\frac{\gamma q_{s}}{h}$$

#### 3.3.2. The Boundary Condition

**Figure 11:**
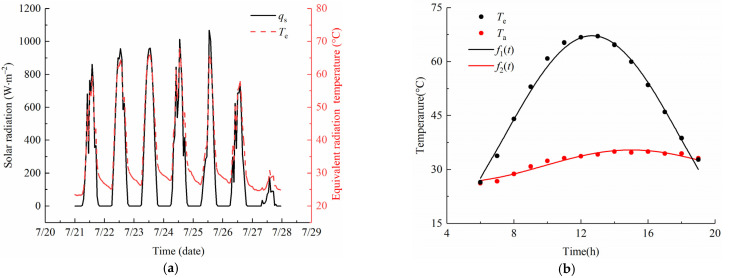
Time history curves of environmental parameters. (**a**) Measured data of the equivalent radiation temperature and solar radiation from 21 to 27 July 2020; (**b**) Fitting results of the equivalent radiation temperature and air temperature on 23 July 2020.

Figure 11a shows the time history curves of the equivalent radiation temperature *T*_e_ and solar radiation *q*_s_ from 21 to 27 July 2020 at the test site. The curve of the equivalent radiation temperature is proportional to that of solar radiation, and the change trends are consistent. The variable *T*_e_ changes regularly with time, which makes it convenient to be fitted by the cosine function (Equation (1)) during the solar time. In addition, the continuous warming weather condition makes the uniform temperature distribution in the concrete slab; thereby, the heating data on the third day were selected to determine the govern equations of the temperature model, as shown in Figure 11b. 

In Figure 11b, the temperature variation of the parameter *T*_e_ exposed to the solar radiation is wider. The fitting results of the equivalent radiation temperature and air temperature have a good degree of correlation, with an index *R*^2^ of 0.99 and 0.98, respectively. The fitting formulas *f*_1_(*t*) and *f*_2_(*t*) at the boundaries are, respectively:

(31) $$f_{1}\left(t\right)=32.9-32.53\cos\left[\left(\frac{2\pi }{23.43}\left(t-0.891\right)\right)\right],R^{2}=0.99$$

(32) $$f_{2}\left(t\right)=31+4.5\cos\left[\left(\frac{2\pi }{20.5}\left(t-15\right)\right)\right],R^{2}=0.98$$

#### 3.3.3. The Initial Condition

The sensitivity for the initial condition may result in the instability of the calculation for the time period in which the temperature gradient is high [18]. To avoid the unexpected oscillations in solutions, the initial temperature distribution in concrete structure was assumed to be uniform and equal to the atmospheric temperature at initial time. Emerson et al. [28] pointed out that the initial temperature can be approximated by the atmospheric temperature at 8:00, which was verified for the temperature of concrete beams and composite beams. The calculated result is more accurate when the initial condition is determined by the measurement of the structural temperature [29,30]. 

**Figure 12:**
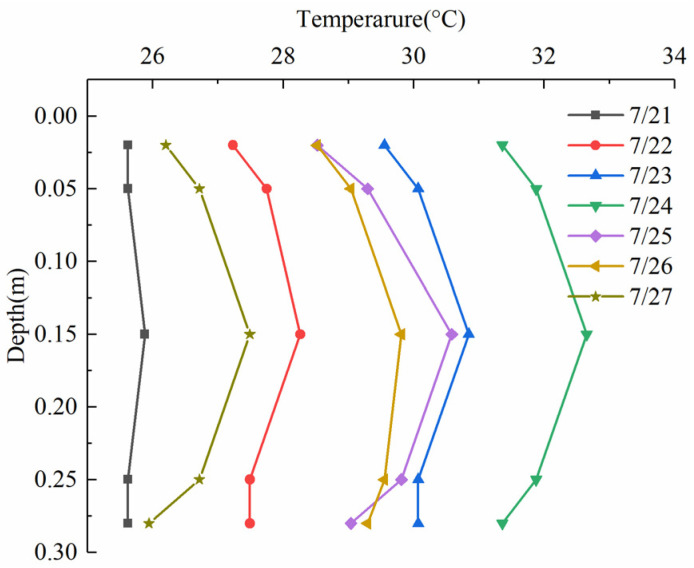
Initial temperature distributions at 6:00 for a week.

Figure 12 shows the initial temperature distributions through the depth of the slab at 6:00 for a week. These temperature profiles present various nonlinear temperature distributions and temperature differences. The expression of the temperature profile is necessary when applying analytical methods. Based on the above assumptions of the initial condition, a uniform temperature along the depth was calculated by the measured temperatures on 23 July. The maximum error between the calculated temperature and the measured one was 0.72 °C. Thereby, the calculated temperature (30 °C) was taken as the initial condition of the temperature model.

#### 3.3.4. The Total Heat Transfer Coefficient

The influence of total heat transfer coefficient on the predicted temperature has been studied and it was shown that the result with the daily average coefficient instead of the time-change coefficient involved a maximum error of 6% [31]. The daily averages of the total heat transfer coefficient on 23 July were calculated using Equation (29), which were *h*_1_ = 15.8 W∙m^−2^·K^−1^ and *h*_2_ = 11.4 W∙m^−2^·K^−1^.

## 4. Results and Discussion

### 4.1. Comparison with the Numerical Solution

The numerical solution of the testing specimen (Figure 3) was calculated using the COMSOL software. A three-dimensional thermal field model was established by considering the actual heat transfer behaviors, including the atmospheric radiation, solar radiation, free convection, and reflected radiation, as shown in Figure 10. The measured meteorological parameters including the solar radiation, ambient temperature, and wind speed, were inputted into the boundary conditions of the temperature model. The measured temperature profile on 23 July was taken as the initial condition. Besides, the ground model was established to calculated the reflected radiation. The material properties and thermal coefficients [21,22,31] are listed in Table 1 and Table 2.

**Figure 13:**
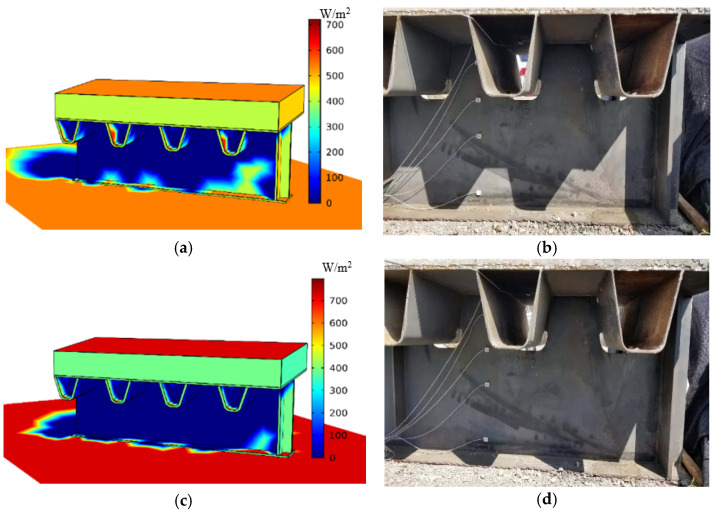
Simulation results of the solar radiation absorbed by surfaces at different times. (**a**) Simulation of the absorbed radiation at 8:00; (**b**) Shading zone at 8:00; (**c**) Simulation of the absorbed radiation at 10:00; (**d**) Shading zone at 10:00.

The simulation results of the solar radiation absorbed by surfaces are shown in Figure 13. Figure 13a,c shows the distribution of solar radiation absorbed by surfaces at 8:00 and 10:00, respectively. The horizontal solar radiation intensity increases from 540 W/m^2^ to 785 W/m^2^ with time variation, and the intensity of the shading area without the solar radiation is 0 W/m^2^. The shading zone matches well that of the testing specimen (Figure 13b,d). This indicates that the simulation method of the thermal field model when considering site conditions is correct.

**Figure 14:**
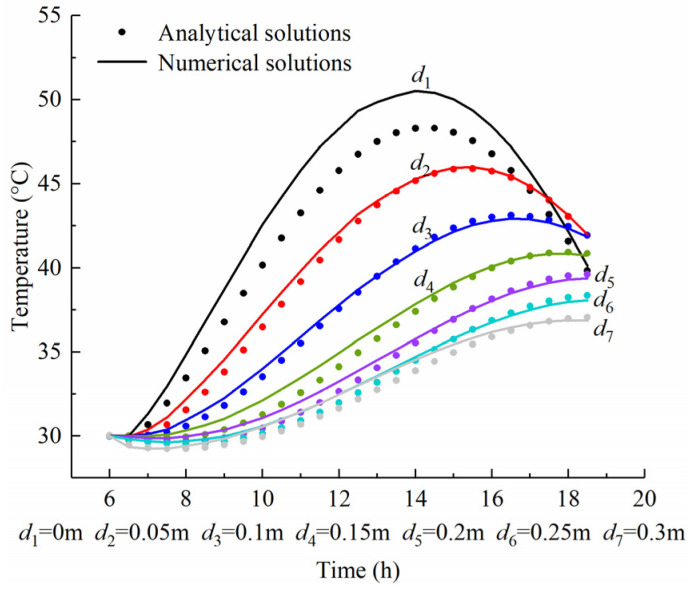
Comparison between numerical solutions and analytical solutions (*n* = 9).

Figure 14 shows the time–temperature curve of the analytical solution (*n* = 9) and numerical solution at different depths. The calculated temperatures at various depths converge to the initial temperature of 30 °C at 6:00. The maximum errors at the top and bottom surface are 2.4 °C and 1.3 °C, respectively. Meanwhile, the error within the concrete slab is less than 0.8 °C. The nonhomogeneous boundary condition makes the nonconvergence of the analytical solution at boundary surfaces. Thereby, the analytical temperature at the boundary surface requires larger superposition terms *n* for generating a higher prediction accuracy.

Table 3 lists the maximum error (*ME*) between the analytical solution and the numerical solution, which is calculated using Equation (33). It can be seen in Table 3 that the convergence at *n* = 1 is rather slow, with an accuracy of 11% reached after four terms. The error after summing the first nine terms is less than 5%. The calculated temperature at *d*_5_ = 0.2 m converged to within 3% (1.3 °C) of the numerical value after summing only the first five terms of the series. In general, as term *n* increases, the convergence of the series summation becomes much more rapid. This is readily explained by the exponential term of Equation (25), which depends on the power of $-\alpha \lambda_{n}^{2}\left(t-t_{1}\right)$. Clearly this term decreases exponentially with increasing time and increasing eigenvalues, thereby causing the rapid convergence of the series.

(33) $$ME=\left\{\begin{array}{l} \max(T(n,x,t)-T_{m}(x,t)/T_{m}(x,t)\times 100\%)\;T(x,t)>T_{m}(x,t)\\ \min(T(n,x,t)-T_{m}(x,t)/T_{m}(x,t)\times 100\%)\;T(x,t)<T_{m}(x,t) \end{array}\right.\;,\;n=1,2,3\cdots 15;\;0<x=d<0.3;\;6<t<19$$

where *T*(*n*, *x*, *t*) is the analytical solution and *T*_m_(*x*, *t*) is the numerical solution.

**Figure 15:**
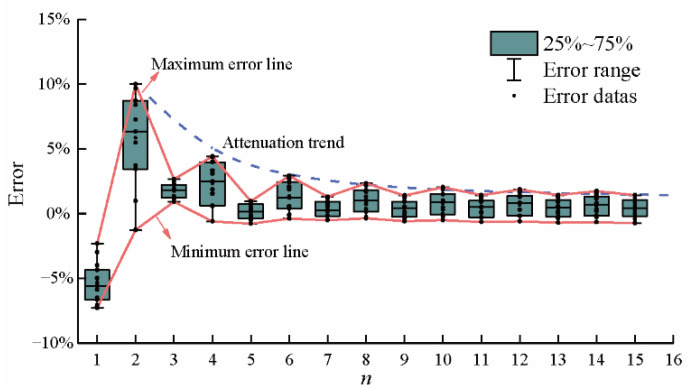
Error results (*d*_7_ = 0.3 m).

The calculation error at the position *d*_7_ = 0.3 m was used for error statistics, which are shown in the boxplot of Figure 15. In Figure 15, the maximum error line clearly fluctuates over a range of *n* = 1 to *n* = 8. With the increase in terms *n*, the fluctuation range of the line is relatively small. When *n* is an odd number, it is beneficial for reducing the error. However, we note that every other term, namely, the even *n* terms, are adverse for convergence. This is readily explained by the positive or negative value of the eigenfunction *X*(*β_n_,x*) in the analytical equation, which depends on the quadrant of the eigenvalue *β_n_*. For the even *n* terms, *β_n_* is in the third quadrant, which produces the positive eigenfunction. *β_n_* changes in the first and third quadrants in turn with the change in the parity of *n*. Thus, the maximum error line clearly fluctuates in first eight terms. Furthermore, the error fluctuation is smaller with greater increases in the terms. This indicates that the errors tend to be stabilized quickly and change linearly.

### 4.2. Comparison with Empirical Formula and Experimental Data

The empirical result of the slab temperature was calculated to present the improvement of the analytical solution. The empirical formula in the one-dimensional temperature model was developed without considering the convection heat transfer at the shading surface. Thereby, the slab temperature is directly related to the equivalent radiation temperature at the radiation surface. The empirical result is calculated by modifying the amplitude and phase of the equivalent radiation temperature. The empirical formula is as follows [15]:

(34) $$T_{f}\left(x,t\right)=f_{1}(t{)}^{\prime }+\gamma \left[f_{1}\left(t-\delta \right)-f_{1}(t{)}^{\prime }\right]\;,\;t_{1}<t<t_{2},\;0<x<d$$

(35) $$\gamma =\frac{\exp\left(-\sqrt{\pi /24\alpha }x\right)}{\sqrt{1+\sqrt{\pi k^{2}/6\alpha h_{u}{}^{2}}+}\pi k^{2}/12\alpha h_{u}{}^{2}}$$

(36) $$\delta =-\tan^{-1}\frac{1}{1+\sqrt{24\alpha h_{u}{}^{2}/\pi k^{2}}}-\sqrt{\pi /24\alpha }x$$

where *f*_1_(*t*)’ is the daily average value of the equivalent radiation temperature *f*_1_(*t*), *γ* is the amplitude correction factor, and *δ* is the phase correction factor.

**Figure 16:**
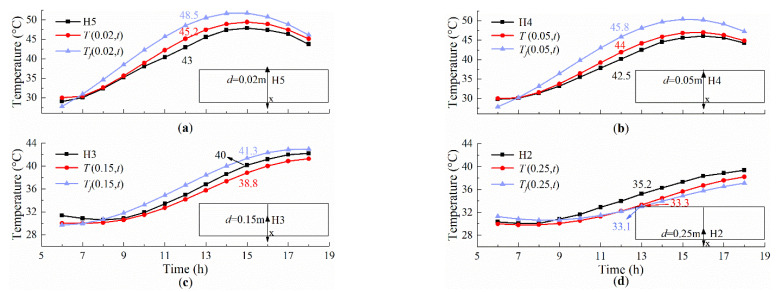
Results of the analytical, empirical, and measured temperatures on 23 July 2020. (**a**) H5; (**b**) H4; (**c**) H3; (**d**) H2.

Figure 16 shows the analytical, empirical, and measured temperatures at different measuring points on 23 July 2020. It can be observed that the analytical result (*T*(*x*, *t*)) matches well with the measured temperature (H2~H5). The errors between the calculated temperature (*T*(*x*, *t*)) and the measured temperature are very small during the three hours before sunset and after sunrise. However, the error becomes larger when the solar radiation is stronger at 10:00~15:00. The maximum error rate of the analytical solution occurs at the measuring point H5, at 5.5% (2.2 °C) in Figure 16a. Only while considering the influence of the top surface on the temperature field, the maximum error rate between the empirical result (*T_f_*(0.02, 12)) and the measured temperature (H5) is 12.8% (5.5 °C) in Figure 16a. This can be explained by the fact that the daily average of total heat transfer coefficient deviates too much from that coefficient, changing with time. By contrast, the analytical method with appropriate assumptions can provide a higher accuracy method for the temperature of the slab track.

### 4.3. Decomposition of the Concrete Slab Surface Temperature

**Figure 17:**
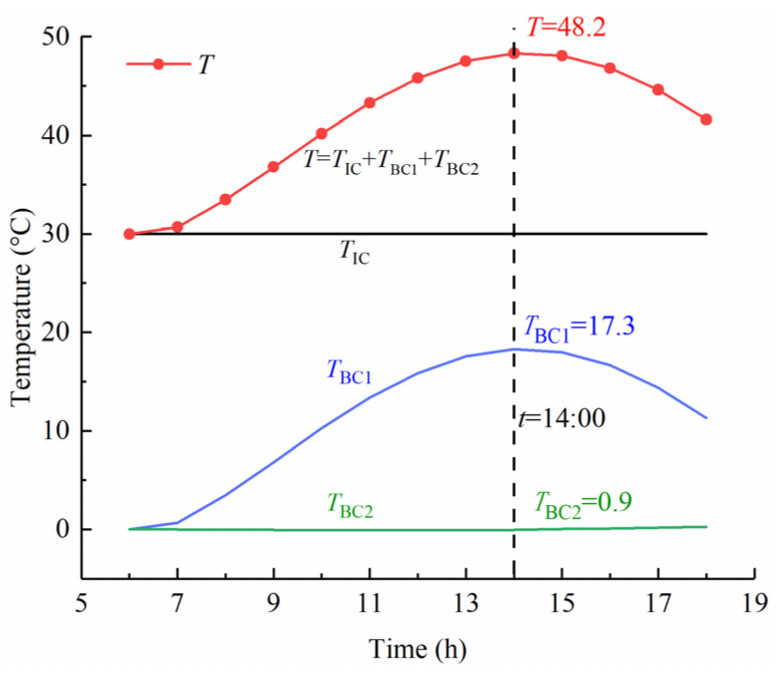
Time history curves of temperature components.

The temperature components of the concrete slab are a function of variables *i*, *x*, and *t*, which can be calculated at an arbitrary point *x* and at any time *t* using Equation (25). Figure 17 shows the time history curve of temperature components at the top slab surface. The curve of component *T*_BC1_ is generated through heat exchange with the solar radiation and air temperature at the top surface (BC1), and the curve of component *T*_BC2_ is generated by convection with the air temperature *f*_2_(*t*) at the bottom surface (BC2).

In the solar radiation duration, the change trend of the surface temperature *T* is consistent with that of the component *T*_BC1_. When time is *t* = 14, the calculated temperature of the top surface is 48.2 °C. Then, by substituting the temperature value into Equations (29) and (25), the instantaneous net heat flux at the top surface is 253 W·m^−^^2^, and the increment of the top surface temperature contributed by the component *T*_BC1_ is 17.3 °C, as shown in Figure 17. In addition, the bottom surface of the slab belongs to the shading surface, where there is only convection heat transfer and a small amount of radiation heat transfer. The limited heat flow (*t* = 14, *q* = 34 W·m^−^^2^) contributes little to the increment in the top surface temperature. The result from the curve of *T*_BC2_ shows that the average contribution of the component *T*_BC2_ is only about 0.5 °C in the daytime.

**Figure 18:**
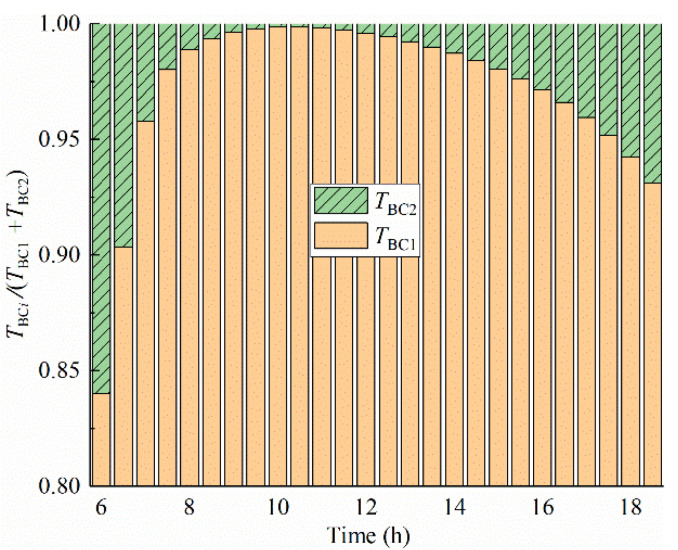
Hourly percentages of components *T*_BC1_ and *T*_BC2_ at the top surface.

Figure 18 shows the hourly percentages of the components *T*_BC1_ and *T*_BC2_ in the heating process of the top surface. According to statistics, the contribution percentage of the bottom surface temperature accounts on average for only 5% in the increment of the top surface temperature. The result shows that the temperature change of the top surface is mainly affected by the temperature components *T*_BC1_. For instance, when the top surface temperature increases by 20 °C, the bottom surface temperature only contributes about 1 °C to the increment in the top surface temperature.

The comparison of the change characteristics of the temperature components *T*_BC1_ and *T*_BC2_ shows that the temperature variation in the top surface is related to the component *T*_BC1_ and is independent of component *T*_BC2_. Using the decomposition method, we can also obtain the similar change trend of temperature components at different depths.

### 4.4. Decomposition of the Temperature Distribution of the Concrete Slab

**Figure 19:**
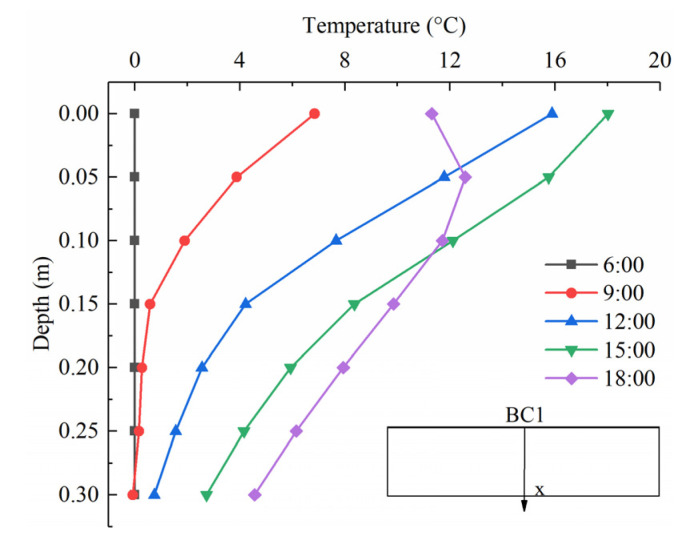
Temperature distributions of component *T*_BC1_ at different times.

Figure 19 illustrates the temperature distribution of the temperature component *T*_BC1_ through the depth of the slab at different times. The temperature profile of the slab is approximately a straight line at 6:00. With the more heat absorbed by the surface BC1, the profile becomes nonlinear over the solar time. The largest temperature variation occurs on the top surface and reaches a maximum of 17.6 °C at 15:00, while the bottom surface temperature is 3.7 °C. After that time, the top surface temperature begins to drop. However, due to the low thermal conductivity of concrete, the bottom surface temperature steadily increases and reaches 5.2 °C at 18:00. The maximum positive temperature difference is 14.7 °C at 15:00 under the thermal action of solar radiation.

**Figure 20:**
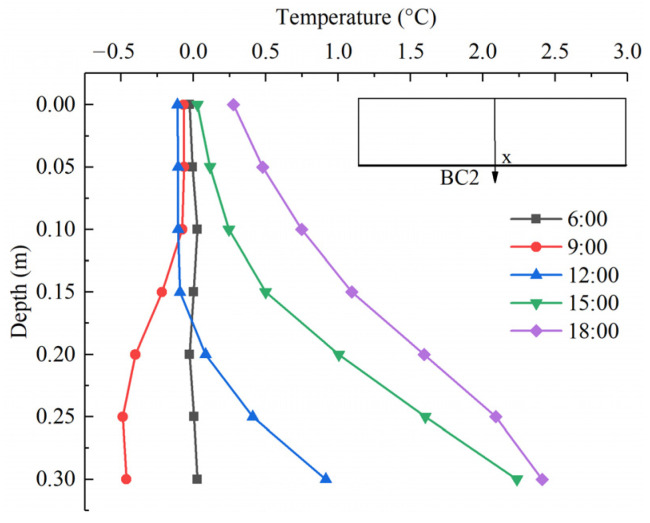
Temperature distributions of component *T*_BC2_ at different times.

Figure 20 shows the temperature distribution of component *T*_BC2_ at different times. Only with the convective and reflected radiation heat transfer at the bottom surface (BC2), the temperature difference varies slightly in the daytime. In addition, one can observe that the negative temperature difference occurs at 6:00~9:00. Due to the weak solar radiation during early sunrise hours, the surface BC2 remains heat loss, and the temperature at a depth of 0.1~0.3 m drops. With the limited amount of heat loss, the negative temperature difference is very small, at just −0.5 °C. The temperature difference values become gradually positive with an increment of the combined action of the convection and reflected radiation on the surface BC2. The maximum positive temperature difference is 2.2 °C at 18:00 under the thermal action of air temperature.

The comparison of the temperature distribution of the temperature component shows that the solar radiation has a great influence on the nonlinear temperature distribution of the concrete slab. Due to the different amount of the heat exchange at the boundary surface, the temperature profiles of the temperature components *T*_BC1_ and *T*_BC2_ are not symmetric along the depth of the concrete slab. 

## 5. Conclusions

With a special focus on the influence of environmental conditions on the temperature distribution of slab tracks, an analytical method is proposed to calculate and decompose the temperature distribution of slab tracks. A one-dimensional temperature model was solved using the integral transformation method. An experimental program on a concrete slab track structure was conducted to validate the reliability and accuracy of the developed methodology in this study. Based on the results, the following conclusions are drawn:

(1) The proposed method is convenient to predict the real-time temperature distribution of concrete slab tracks using meteorological parameters, and shows a high accuracy and a rapid convergence speed.
(2) The relationship between the temperature of the slab track and meteorological parameters is established through the proposed analytical solution. Based on the temperature decomposition method, the temperature distribution of slab tracks affected by solar radiation and atmospheric temperature can be calculated separately.
(3) A method for dealing with meteorological parameters is proposed. The combined action of solar radiation and atmospheric temperature on the boundary surface is considered as a fluid medium, which is the expression of a cosine function.
(4) Solar radiation is the main reason for the nonlinear temperature distribution in slab tracks during the daytime. By contrast, the convection heat transfer caused by air has little effect, and the temperature change in the slab surface resulting from the atmospheric temperature accounts for only 5% in the hot weather condition.

In this study, an analytical method of the one-dimensional thermal field in a slab track is proposed. To improve the applicability of the method, a multidimensional temperature model of slab tracks in different environmental conditions need to be investigated. In future works, the transverse temperature distribution can be considered in temperature measurement, and the effect of extreme weather conditions on slab tracks can be further studied.

## Figures and Tables

**Table 1:** Properties of concrete and steel.

Material Property	Concrete	Steel
Density, *ρ* (Kg·m^−3^)	2800	7850
Specific heat capacity, *c* (J·Kg^−1^·K^−1^)	880	475
Thermal conductivity, *k* (W m^−1^·K^−1^)	1.8	47

**Table 2:** Thermal coefficients adopted in the temperature model.

Thermal Coefficient	Concrete	Steel
Shortwave absorptivity, *γ* (W m^−1^·K^−1^)	0.5	0.9
Longwave absorptivity, *γ*_1_ (W m^−1^·K^−1^)	0.82	0.88
Emissivity, *ε*	0.82	0.88

**Table 3:** Error statistical results.

*N*	Maximum Error						
	*d*_1_ = 0 m	*d*_2_ = 0.05 m	*d*_3_ = 0.1 m	*d*_4_ = 0.15 m	*d*_5_ = 0.2 m	*d*_6_ = 0.25 m	*d*_7_ = 0.3 m
1	30.46%	16.99%	4.60%	5.59%	11.25%	11.80%	6.96%
2	20.24%	3.6%	6.55%	6.90%	4.92%	6.91%	10.17%
3	13.79%	2.95%	2.26%	4.11%	2.03%	2.41%	2.66%
4	10.73%	3.37%	3.69%	4.24%	2.95%	3.83%	4.54%
5	8.56%	2.63%	3.01%	2.59%	1.96%	2.21%	2.55%
6	7.26%	3.09%	3.31%	2.61%	2.35%	2.42%	2.96%
7	6.26%	2.31%	1.84%	1.27%	1.46%	2.17%	2.42%
8	5.61%	2.45%	1.97%	1.32%	1.54%	1.95%	2.54%
9	4.34%	1.93%	1.64%	1.15%	1.22%	1.52%	2.1%

## Data Availability

The data that support the findings of this study are available upon request from the authors.
